# 2-Phenyl-2,3-dihydro­phenanthro[9,10-*b*][1,4]dioxine

**DOI:** 10.1107/S1600536811003904

**Published:** 2011-02-05

**Authors:** Hoong-Kun Fun, Ching Kheng Quah, Dongdong Wu, Yan Zhang

**Affiliations:** a X-ray Crystallography Unit, School of Physics, Universiti Sains Malaysia, 11800 USM, Penang, Malaysia; b School of Chemistry and Chemical Engineering, Nanjing University, Nanjing 210093, People’s Republic of China

## Abstract

In the title compound, C_22_H_16_O_2_, the phenanthrene ring system is essentially planar [maximum deviation = 0.058 (1) Å] and is inclined at an angle of 58.39 (6)° to the phenyl ring. The 1,4-dioxane ring is in a chair conformation. In the crystal, mol­ecules are stacked along the *b* axis, but no significant hydrogen bonds are observed.

## Related literature

For general background to and details of the biological activity of phenanthrene derivatives, see: Wang *et al.* (2010 ▶); Li & Wang (2009 ▶); Gao & Wong (2010 ▶); Zhan & Jiang (2010 ▶); Becker & Dettbarn (2009 ▶); Jones & Mathews (1997 ▶). For ring conformations, see: Cremer & Pople (1975 ▶). For bond-length data, see: Allen *et al.* (1987 ▶).
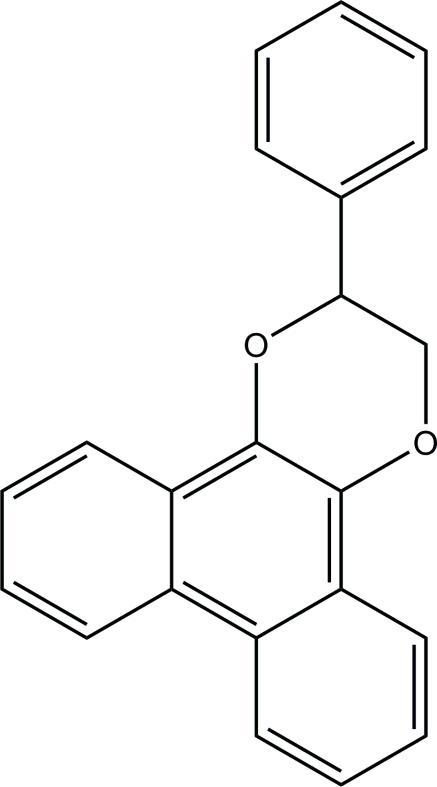


## Experimental

### 

#### Crystal data

C_22_H_16_O_2_
*M* *_r_* = 312.35
Monoclinic, 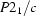

*a* = 12.1831 (3) Å
*b* = 5.4674 (1) Å
*c* = 24.6064 (7) Å
β = 106.005 (2)°
*V* = 1575.50 (7) Å^3^
*Z* = 4
Mo *K*α radiation
μ = 0.08 mm^−1^
*T* = 296 K
0.49 × 0.41 × 0.13 mm

#### Data collection

Bruker SMART APEXII CCD area-detector diffractometer
Absorption correction: multi-scan (*SADABS*; Bruker, 2009 ▶) *T*_min_ = 0.960, *T*_max_ = 0.989
17018 measured reflections
4613 independent reflections
2927 reflections with *I* > 2σ(*I*)
*R*_int_ = 0.030

#### Refinement

*R*[*F* ^2^ > 2σ(*F* ^2^)] = 0.049
*wR*(*F* ^2^) = 0.145
*S* = 1.04
4613 reflections
217 parameters
H-atom parameters constrained
Δρ_max_ = 0.13 e Å^−3^
Δρ_min_ = −0.18 e Å^−3^

### 

Data collection: *APEX2* (Bruker, 2009 ▶); cell refinement: *SAINT* (Bruker, 2009 ▶); data reduction: *SAINT*; program(s) used to solve structure: *SHELXTL* (Sheldrick, 2008 ▶); program(s) used to refine structure: *SHELXTL*; molecular graphics: *SHELXTL*; software used to prepare material for publication: *SHELXTL* and *PLATON* (Spek, 2009 ▶).

## Supplementary Material

Crystal structure: contains datablocks global, I. DOI: 10.1107/S1600536811003904/sj5097sup1.cif

Structure factors: contains datablocks I. DOI: 10.1107/S1600536811003904/sj5097Isup2.hkl

Additional supplementary materials:  crystallographic information; 3D view; checkCIF report

## Figures and Tables

**Fig. 1.**
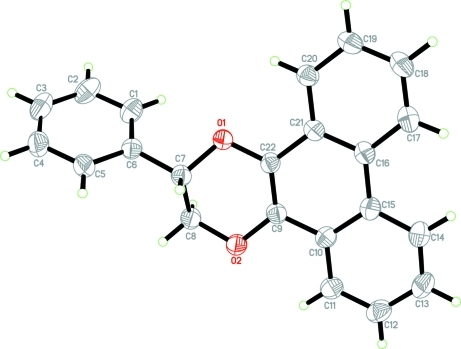
The asymmetric unit of the title compound showing 30% probability displacement ellipsoids for non-H atoms.

**Fig. 2.**
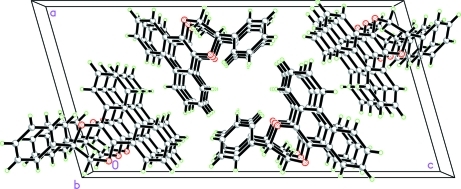
The crystal structure of the title compound, viewed along the b axis.

## supplementary crystallographic information

### Comment

Phenanthrene is a major structural component of many natural products that play important roles in pharmaceutical and biological fields. For example, phenanthrene-based tylophorine derivatives could serve as potential antiviral agents against the tobacco mosaic virus in vitro (Wang *et al.*, 2010). In addition, phenanthrene-based alkaloids are found to possess antitumor activities (Li & Wang, 2009) and other important bioactivities. Gao & Wong reported that phenanthrene has important effect on rice cultivation by degrading the bacterium affecting the rice plants (Gao & Wong, 2010). In a phenanthrene-contaminated soil, the activity of urease and catalase may be decreased while polyphenol oxidase was stimulated (Zhan & Jiang, 2010). The importance of 9,10-disubstituted phenanthrene in biochemistry also has been reported. Phenanthrene 9,10-dihydrodiol could be used as a biomarker for ETS-exposure of children and the derivatives of pyrrolo(9, 10b)-phenanthrene were good templates for DNA intercalating drug delivery system (Becker & Dettbarn, 2009; Jones & Mathews, 1997). Due to the importance of the 9, 10-disubstituted phenanthrene derivatives, herewith, we report the crystal structure of the title compound.

The title compound (Fig. 1) is made up of a phenanthrene (C9-C22) ring system, a phenyl (C1-C6) ring and a 1,4-dioxane (O1/O2/C7-C9/C22) ring. The phenanthrene ring system is essentially planar, with a maximum deviation of 0.058 (1) Å at atom C21, and is inclined at an angle of 58.39 (6) ° with the phenyl ring. The 1,4-dioxane ring is in chair conformation, puckering parameters (Cremer & Pople, 1975) Q = 0.4811 (14) Å; Θ = 51.41 (16)° and φ = 79.22 (18)°. Bond lengths (Allen *et al.*, 1987) and angles are within normal ranges. In the crystal (Fig.2), molecules are stacked along the *b*-axis but no significant hydrogen bonds are observed.

### Experimental

The title compound is a product of the photoreaction between phenanthrenequinone and styrene. The compound was purified by flash column chromatography with ethyl acetate/petroleum ether (1:10) as eluents. Good quality crystals of the title compound were obtained from slow evaporation of an acetone and petroleum ether solution (1:10).

### Refinement

All H atoms were positioned geometrically and refined using a riding model with C–H = 0.93 -0.98 Å and *U*_iso_(H) = 1.2 *U*_eq_(C). The highest residual electron density peak is located at 0.74 Å from H1A and the deepest hole is located at 1.33 Å from C14.

### Crystal data

**Table:**

C_22_H_16_O_2_	*F*(000) = 656
*M**_r_* = 312.35	*D*_x_ = 1.317 Mg m^−^^3^
Monoclinic, *P*2_1_/*c*	Mo *K*α radiation, λ = 0.71073 Å
Hall symbol: -P 2ybc	Cell parameters from 4055 reflections
*a* = 12.1831 (3) Å	θ = 2.8–28.1°
*b* = 5.4674 (1) Å	µ = 0.08 mm^−^^1^
*c* = 24.6064 (7) Å	*T* = 296 K
β = 106.005 (2)°	Plate, colourless
*V* = 1575.50 (7) Å^3^	0.49 × 0.41 × 0.13 mm
*Z* = 4	

### Data collection

**Table:**

Bruker SMART APEXII CCD area-detector diffractometer	4613 independent reflections
Radiation source: fine-focus sealed tube	2927 reflections with *I* > 2σ(*I*)
graphite	*R*_int_ = 0.030
φ and ω scans	θ_max_ = 30.1°, θ_min_ = 2.1°
Absorption correction: multi-scan (*SADABS*; Bruker, 2009)	*h* = −17→17
*T*_min_ = 0.960, *T*_max_ = 0.989	*k* = −7→7
17018 measured reflections	*l* = −34→34

### Refinement

**Table:**

Refinement on *F*^2^	Primary atom site location: structure-invariant direct methods
Least-squares matrix: full	Secondary atom site location: difference Fourier map
*R*[*F*^2^ > 2σ(*F*^2^)] = 0.049	Hydrogen site location: inferred from neighbouring sites
*wR*(*F*^2^) = 0.145	H-atom parameters constrained
*S* = 1.04	*w* = 1/[σ^2^(*F*_o_^2^) + (0.0666*P*)^2^ + 0.1135*P*] where *P* = (*F*_o_^2^ + 2*F*_c_^2^)/3
4613 reflections	(Δ/σ)_max_ = 0.001
217 parameters	Δρ_max_ = 0.13 e Å^−^^3^
0 restraints	Δρ_min_ = −0.18 e Å^−^^3^

### Special details

**Table:**

Geometry. All esds (except the esd in the dihedral angle between two l.s. planes) are estimated using the full covariance matrix. The cell esds are taken into account individually in the estimation of esds in distances, angles and torsion angles; correlations between esds in cell parameters are only used when they are defined by crystal symmetry. An approximate (isotropic) treatment of cell esds is used for estimating esds involving l.s. planes.
Refinement. Refinement of F^2^ against ALL reflections. The weighted R-factor wR and goodness of fit S are based on F^2^, conventional R-factors R are based on F, with F set to zero for negative F^2^. The threshold expression of F^2^ > 2sigma(F^2^) is used only for calculating R-factors(gt) etc. and is not relevant to the choice of reflections for refinement. R-factors based on F^2^ are statistically about twice as large as those based on F, and R- factors based on ALL data will be even larger.

### Fractional atomic coordinates and isotropic or equivalent isotropic displacement parameters (Å^2^)

**Table:**

	*x*	*y*	*z*	*U*_iso_*/*U*_eq_	
O1	0.69332 (7)	0.38054 (17)	0.93970 (4)	0.0546 (2)	
O2	0.88773 (8)	0.39205 (19)	0.89709 (4)	0.0634 (3)	
C1	0.70209 (14)	−0.0387 (3)	1.00451 (6)	0.0650 (4)	
H1A	0.6455	−0.0275	0.9704	0.078*	
C2	0.69661 (18)	−0.2210 (3)	1.04270 (7)	0.0778 (5)	
H2A	0.6366	−0.3325	1.0340	0.093*	
C3	0.77931 (18)	−0.2381 (3)	1.09335 (7)	0.0790 (5)	
H3A	0.7755	−0.3612	1.1189	0.095*	
C4	0.86718 (15)	−0.0739 (3)	1.10617 (7)	0.0779 (5)	
H4A	0.9232	−0.0854	1.1405	0.093*	
C5	0.87316 (13)	0.1090 (3)	1.06854 (6)	0.0643 (4)	
H5A	0.9327	0.2215	1.0778	0.077*	
C6	0.79093 (11)	0.1262 (2)	1.01686 (5)	0.0502 (3)	
C7	0.80427 (11)	0.3130 (2)	0.97448 (5)	0.0504 (3)	
H7A	0.8427	0.4579	0.9944	0.060*	
C8	0.87275 (12)	0.2128 (3)	0.93669 (6)	0.0590 (3)	
H8A	0.9470	0.1598	0.9598	0.071*	
H8B	0.8338	0.0716	0.9164	0.071*	
C9	0.79160 (10)	0.5302 (2)	0.87464 (5)	0.0501 (3)	
C10	0.79662 (11)	0.6914 (2)	0.82942 (5)	0.0505 (3)	
C11	0.89116 (12)	0.6902 (3)	0.80716 (6)	0.0619 (4)	
H11A	0.9501	0.5790	0.8208	0.074*	
C12	0.89711 (14)	0.8519 (3)	0.76553 (6)	0.0706 (4)	
H12A	0.9596	0.8491	0.7507	0.085*	
C13	0.81042 (15)	1.0190 (3)	0.74551 (6)	0.0706 (4)	
H13A	0.8158	1.1311	0.7179	0.085*	
C14	0.71683 (14)	1.0214 (3)	0.76584 (6)	0.0639 (4)	
H14A	0.6590	1.1344	0.7516	0.077*	
C15	0.70603 (11)	0.8569 (2)	0.80781 (5)	0.0511 (3)	
C16	0.60599 (11)	0.8495 (2)	0.82931 (5)	0.0500 (3)	
C17	0.51242 (13)	1.0099 (3)	0.81093 (6)	0.0629 (4)	
H17A	0.5139	1.1283	0.7840	0.075*	
C18	0.41948 (13)	0.9955 (3)	0.83177 (6)	0.0669 (4)	
H18A	0.3593	1.1047	0.8192	0.080*	
C19	0.41436 (12)	0.8191 (3)	0.87158 (6)	0.0626 (4)	
H19A	0.3503	0.8082	0.8850	0.075*	
C20	0.50375 (11)	0.6612 (3)	0.89093 (6)	0.0545 (3)	
H20A	0.5000	0.5429	0.9175	0.065*	
C21	0.60128 (10)	0.6763 (2)	0.87100 (5)	0.0473 (3)	
C22	0.69876 (10)	0.5239 (2)	0.89464 (5)	0.0479 (3)	

### Atomic displacement parameters (Å^2^)

**Table:**

	*U*^11^	*U*^22^	*U*^33^	*U*^12^	*U*^13^	*U*^23^
O1	0.0505 (5)	0.0640 (5)	0.0521 (5)	0.0110 (4)	0.0191 (4)	0.0106 (4)
O2	0.0592 (6)	0.0747 (6)	0.0649 (6)	0.0212 (5)	0.0314 (5)	0.0178 (5)
C1	0.0845 (10)	0.0617 (8)	0.0518 (7)	−0.0079 (7)	0.0238 (7)	−0.0085 (7)
C2	0.1138 (13)	0.0596 (8)	0.0736 (10)	−0.0131 (9)	0.0484 (10)	−0.0079 (8)
C3	0.1175 (15)	0.0642 (9)	0.0705 (10)	0.0252 (10)	0.0512 (10)	0.0158 (8)
C4	0.0789 (11)	0.0925 (12)	0.0645 (9)	0.0307 (10)	0.0234 (8)	0.0236 (9)
C5	0.0577 (8)	0.0761 (9)	0.0595 (8)	0.0153 (7)	0.0166 (6)	0.0094 (7)
C6	0.0571 (7)	0.0507 (7)	0.0471 (6)	0.0110 (6)	0.0216 (6)	−0.0017 (5)
C7	0.0521 (7)	0.0520 (7)	0.0476 (7)	0.0079 (5)	0.0147 (5)	0.0002 (5)
C8	0.0630 (8)	0.0620 (8)	0.0576 (8)	0.0183 (6)	0.0258 (6)	0.0086 (7)
C9	0.0507 (7)	0.0542 (7)	0.0468 (6)	0.0081 (5)	0.0160 (5)	−0.0011 (6)
C10	0.0575 (7)	0.0538 (7)	0.0424 (6)	−0.0003 (6)	0.0173 (5)	−0.0052 (5)
C11	0.0644 (9)	0.0720 (9)	0.0546 (8)	0.0038 (7)	0.0255 (6)	0.0002 (7)
C12	0.0743 (10)	0.0852 (11)	0.0600 (8)	−0.0074 (8)	0.0315 (7)	0.0007 (8)
C13	0.0871 (11)	0.0733 (9)	0.0535 (8)	−0.0100 (9)	0.0229 (8)	0.0090 (7)
C14	0.0744 (9)	0.0633 (8)	0.0523 (7)	0.0020 (7)	0.0147 (7)	0.0060 (7)
C15	0.0603 (8)	0.0519 (7)	0.0394 (6)	−0.0017 (6)	0.0108 (5)	−0.0051 (5)
C16	0.0543 (7)	0.0515 (7)	0.0410 (6)	0.0038 (5)	0.0078 (5)	−0.0060 (5)
C17	0.0700 (9)	0.0598 (8)	0.0545 (8)	0.0120 (7)	0.0098 (7)	0.0025 (7)
C18	0.0609 (9)	0.0694 (9)	0.0664 (9)	0.0197 (7)	0.0106 (7)	−0.0024 (8)
C19	0.0508 (8)	0.0738 (9)	0.0620 (8)	0.0089 (7)	0.0136 (6)	−0.0084 (7)
C20	0.0506 (7)	0.0608 (8)	0.0518 (7)	0.0042 (6)	0.0139 (6)	−0.0028 (6)
C21	0.0477 (7)	0.0501 (6)	0.0425 (6)	0.0027 (5)	0.0095 (5)	−0.0068 (5)
C22	0.0522 (7)	0.0505 (6)	0.0419 (6)	0.0048 (5)	0.0147 (5)	0.0002 (5)

### Geometric parameters (Å, °)

**Table:**

O1—C22	1.3742 (14)	C10—C11	1.4053 (19)
O1—C7	1.4347 (15)	C10—C15	1.4130 (18)
O2—C9	1.3749 (15)	C11—C12	1.370 (2)
O2—C8	1.4285 (16)	C11—H11A	0.9300
C1—C6	1.376 (2)	C12—C13	1.381 (2)
C1—C2	1.384 (2)	C12—H12A	0.9300
C1—H1A	0.9300	C13—C14	1.366 (2)
C2—C3	1.373 (3)	C13—H13A	0.9300
C2—H2A	0.9300	C14—C15	1.4027 (18)
C3—C4	1.366 (3)	C14—H14A	0.9300
C3—H3A	0.9300	C15—C16	1.4570 (19)
C4—C5	1.378 (2)	C16—C21	1.4086 (18)
C4—H4A	0.9300	C16—C17	1.4103 (18)
C5—C6	1.3882 (19)	C17—C18	1.368 (2)
C5—H5A	0.9300	C17—H17A	0.9300
C6—C7	1.5002 (18)	C18—C19	1.388 (2)
C7—C8	1.5128 (18)	C18—H18A	0.9300
C7—H7A	0.9800	C19—C20	1.3680 (18)
C8—H8A	0.9700	C19—H19A	0.9300
C8—H8B	0.9700	C20—C21	1.4074 (18)
C9—C22	1.3527 (17)	C20—H20A	0.9300
C9—C10	1.4337 (17)	C21—C22	1.4361 (17)
			
C22—O1—C7	112.41 (9)	C15—C10—C9	119.32 (11)
C9—O2—C8	113.33 (10)	C12—C11—C10	120.44 (14)
C6—C1—C2	120.25 (15)	C12—C11—H11A	119.8
C6—C1—H1A	119.9	C10—C11—H11A	119.8
C2—C1—H1A	119.9	C11—C12—C13	120.06 (14)
C3—C2—C1	120.32 (17)	C11—C12—H12A	120.0
C3—C2—H2A	119.8	C13—C12—H12A	120.0
C1—C2—H2A	119.8	C14—C13—C12	120.62 (14)
C4—C3—C2	119.81 (15)	C14—C13—H13A	119.7
C4—C3—H3A	120.1	C12—C13—H13A	119.7
C2—C3—H3A	120.1	C13—C14—C15	121.39 (14)
C3—C4—C5	120.33 (16)	C13—C14—H14A	119.3
C3—C4—H4A	119.8	C15—C14—H14A	119.3
C5—C4—H4A	119.8	C14—C15—C10	117.76 (12)
C4—C5—C6	120.40 (16)	C14—C15—C16	122.88 (12)
C4—C5—H5A	119.8	C10—C15—C16	119.36 (11)
C6—C5—H5A	119.8	C21—C16—C17	117.38 (12)
C1—C6—C5	118.87 (13)	C21—C16—C15	119.29 (11)
C1—C6—C7	121.44 (12)	C17—C16—C15	123.32 (12)
C5—C6—C7	119.58 (13)	C18—C17—C16	121.56 (14)
O1—C7—C6	108.98 (10)	C18—C17—H17A	119.2
O1—C7—C8	108.36 (10)	C16—C17—H17A	119.2
C6—C7—C8	111.39 (10)	C17—C18—C19	120.48 (13)
O1—C7—H7A	109.4	C17—C18—H18A	119.8
C6—C7—H7A	109.4	C19—C18—H18A	119.8
C8—C7—H7A	109.4	C20—C19—C18	119.84 (14)
O2—C8—C7	111.52 (11)	C20—C19—H19A	120.1
O2—C8—H8A	109.3	C18—C19—H19A	120.1
C7—C8—H8A	109.3	C19—C20—C21	120.61 (13)
O2—C8—H8B	109.3	C19—C20—H20A	119.7
C7—C8—H8B	109.3	C21—C20—H20A	119.7
H8A—C8—H8B	108.0	C20—C21—C16	120.07 (11)
C22—C9—O2	123.01 (11)	C20—C21—C22	120.54 (11)
C22—C9—C10	121.12 (11)	C16—C21—C22	119.33 (11)
O2—C9—C10	115.82 (11)	C9—C22—O1	122.63 (11)
C11—C10—C15	119.68 (12)	C9—C22—C21	121.26 (11)
C11—C10—C9	120.98 (12)	O1—C22—C21	116.06 (10)
			
C6—C1—C2—C3	0.4 (2)	C11—C10—C15—C14	−2.33 (19)
C1—C2—C3—C4	0.2 (2)	C9—C10—C15—C14	176.42 (11)
C2—C3—C4—C5	0.0 (2)	C11—C10—C15—C16	177.24 (12)
C3—C4—C5—C6	−0.9 (2)	C9—C10—C15—C16	−4.02 (18)
C2—C1—C6—C5	−1.3 (2)	C14—C15—C16—C21	179.16 (12)
C2—C1—C6—C7	175.03 (13)	C10—C15—C16—C21	−0.38 (17)
C4—C5—C6—C1	1.5 (2)	C14—C15—C16—C17	−1.50 (19)
C4—C5—C6—C7	−174.83 (13)	C10—C15—C16—C17	178.96 (12)
C22—O1—C7—C6	−171.11 (9)	C21—C16—C17—C18	−1.3 (2)
C22—O1—C7—C8	−49.75 (14)	C15—C16—C17—C18	179.35 (13)
C1—C6—C7—O1	31.63 (16)	C16—C17—C18—C19	−0.7 (2)
C5—C6—C7—O1	−152.09 (11)	C17—C18—C19—C20	1.2 (2)
C1—C6—C7—C8	−87.87 (15)	C18—C19—C20—C21	0.2 (2)
C5—C6—C7—C8	88.41 (15)	C19—C20—C21—C16	−2.18 (19)
C9—O2—C8—C7	−40.45 (16)	C19—C20—C21—C22	174.94 (12)
O1—C7—C8—O2	61.41 (15)	C17—C16—C21—C20	2.68 (18)
C6—C7—C8—O2	−178.73 (11)	C15—C16—C21—C20	−177.94 (11)
C8—O2—C9—C22	10.01 (18)	C17—C16—C21—C22	−174.49 (11)
C8—O2—C9—C10	−172.32 (11)	C15—C16—C21—C22	4.90 (17)
C22—C9—C10—C11	−177.33 (12)	O2—C9—C22—O1	0.8 (2)
O2—C9—C10—C11	4.95 (18)	C10—C9—C22—O1	−176.74 (11)
C22—C9—C10—C15	3.94 (19)	O2—C9—C22—C21	178.22 (11)
O2—C9—C10—C15	−173.77 (11)	C10—C9—C22—C21	0.67 (19)
C15—C10—C11—C12	1.4 (2)	C7—O1—C22—C9	21.05 (16)
C9—C10—C11—C12	−177.34 (13)	C7—O1—C22—C21	−156.48 (10)
C10—C11—C12—C13	0.6 (2)	C20—C21—C22—C9	177.71 (12)
C11—C12—C13—C14	−1.6 (2)	C16—C21—C22—C9	−5.14 (18)
C12—C13—C14—C15	0.6 (2)	C20—C21—C22—O1	−4.72 (17)
C13—C14—C15—C10	1.4 (2)	C16—C21—C22—O1	172.42 (10)
C13—C14—C15—C16	−178.18 (13)		
